# RETRACTION: Effect of Wound Irrigation on the Prevention of Surgical Site Infections: A Meta‐Analysis

**DOI:** 10.1111/iwj.70152

**Published:** 2024-12-05

## Abstract

**RETRACTION:** FuC. , MengL. , MaM. , LiN. , and ZhangJ. , “Effect of Wound Irrigation on the Prevention of Surgical Site Infections: A Meta‐Analysis,” International Wound Journal 19, no. 7 (2022): 1878–1886, 10.1111/iwj.13794.35293119 PMC9615282

The above article, published online on 16 March 2022, in Wiley Online Library (http://onlinelibrary.wiley.com/), has been retracted by agreement between the journal Editor in Chief, Professor Keith Harding; and John Wiley & Sons Ltd. Following an investigation by the publisher, all parties have concluded that this article was accepted solely on the basis of a compromised peer review process. The editors have therefore decided to retract the article. The authors did not respond to our notice regarding the retraction.

**RETRACTION:** C. Fu , L. Meng , M. Ma , N. Li , and J. Zhang , “Effect of Wound Irrigation on the Prevention of Surgical Site Infections: A Meta‐Analysis,” International Wound Journal 19, no. 7 (2022): 1878–1886, 10.1111/iwj.13794.35293119 PMC9615282

The above article, published online on 16 March 2022, in Wiley Online Library (http://onlinelibrary.wiley.com/), has been retracted by agreement between the journal Editor in Chief, Professor Keith Harding; and John Wiley & Sons Ltd. Following an investigation by the publisher, all parties have concluded that this article was accepted solely on the basis of a compromised peer review process. The editors have therefore decided to retract the article. The authors did not respond to our notice regarding the retraction.

